# Flap perfusion of superficial split flap design for guided bone regeneration: A case report with ultrasonography

**DOI:** 10.1002/cap.70053

**Published:** 2026-03-04

**Authors:** Oscar Durán‐Garnica, Karen Villarreal‐Arizpe, Amanda Rodríguez, Oliver Kripfgans, Hsun‐Liang Chan

**Affiliations:** ^1^ Division of Periodontology The Ohio State University College of Dentistry Columbus Ohio USA; ^2^ Department of Periodontology University of Illinois Chicago College of Dentistry Chicago Illinois USA; ^3^ Department of Radiology University of Michigan Ann Arbor Michigan USA

**Keywords:** flap design, jawbone, microsurgery, ridge augmentation, ultrasonography, wound healing

## Abstract

**Background:**

Effective flap management is crucial for successful bone regeneration procedures. Traditional flap release has been performed by the deep split design; recently, the superficial split design revives for its proposed anatomical and biomechanical advantages. To understand the risk of this new flap management design on flap vitality, the aim of this case report is to investigate the perfusion of the flap with ultrasound.

**Methods:**

A single patient with an edentulous ridge exhibiting a horizontal bone deficiency underwent a regenerative procedure utilizing the superficial split technique (Secured Anatomy‐Driven Flap Extension [SAFE] technique). Ultrasonography assessments were conducted before the surgery (baseline [BL]) and at days (D) 3, 10, 21, and at 5 months (MO) post‐surgery. They included brightness‐mode and color velocity/power (CV/CP) at the buccal flap. CV and CP cine loops (videos) were recorded to assess tissue perfusion by surrogate. Three distinct regions of interest were selected, i.e., keratinized mucosa (KM), lining mucosa (LM), and muscle (M).

**Results:**

KM perfusion (CV) increased significantly at D3, then decreased at D10 and D21. The 5MO value was insignificant from BL. LM perfusion non‐significantly decreased at D3 and D10, and returned to BL at D21 and 5MO. Muscle perfusion showed a nonsignificant decreasing trend. CP showed nonsignificant changes post‐surgery relative to BL for the three regions.

**Conclusion:**

Ultrasound could be able to longitudinally quantify post‐surgery tissue perfusion with sufficient spatial resolution to assess KM, LM, and M separately. This pilot ultrasonography study may ease the concern that the superficial split flap design jeopardizes flap vitality.

**Key points:**

- Preliminary ultrasound data suggested that the Secured Anatomy‐Driven Flap Extension (SAFE) technique, based on the superficial split approach, does not jeopardize flap vitality.
- Ultrasound may be a valuable tool for monitoring oral wound healing and guiding treatment decisions.

**Plain language summary:**

Ultrasound imaging technology, being a superior modality for evaluating soft tissue characteristics and able to quantify blood perfusion, is becoming a promising research tool to study oral wound healing. This case report used dental ultrasound to monitor the blood flow of an oral wound for 5 months after a jawbone augmentation procedure. Ultrasound showed gum blood flow peaked at day 3 after the surgery and decreased exponentially until 5 months. Mucosa and muscle may have various blood perfusion recovery patterns than gum tissues that deserve further investigation.

## INTRODUCTION

Post‐extraction ridge atrophy compromises the site for prosthetically driven implant therapy,^1^, ^2^ often necessitating an augmentation procedure.^3^ Since the late 1980s, guided bone regeneration (GBR) has been a widely‐documented ridge augmentation procedure, with a mean horizontal bone gain of approximately 3 mm.^4^, ^5^ Despite generally satisfactory results, postoperative complications, e.g., wound dehiscence, are common (16.8%), decreasing the amount of bone gain.^6^, ^7^, ^8^ Therefore, tension‐free wound closure is the first crucial step toward a predictable outcome.^9^

To achieve passive closure, periosteal and vertical releasing incisions are the main techniques.^10^ In a controlled setting, periosteal‐releasing incision allows flap advancement up to 5.5 mm.^11^ Traditional PRI, and its modifications involve deep splitting of the mucosa while preserving a thin periosteal layer so most of the soft tissue, including muscle fibers, is on the flap side.^12^, ^13^, ^14^, ^15^ This approach may also involve dissection of the muscle layer for more flap advancement.^16^, ^17^ Instruments like scissors,^18^ a scalpel with “brushing technique”,^13^ and a blunt‐end instrument^15^, ^19^ are commonly used. However, these methods could increase the incidence of complications such as uncontrolled bleeding, edema, hematoma, and paresthesia from vascular and nerve damage.^13^, ^18^ Additionally, including muscle fibers in the flap may retract during healing, increasing the risk of soft tissue dehiscence.^20^, ^21^ Alternatively, the superficial split technique involves dividing the flap between submucosal connective tissue fibers and muscle.^20^, ^22^ By excluding muscle layers in the flap, muscle pull is minimized, reducing flap retraction.^20^ Nevertheless, the main concern about superficial splitting is the potential to compromise flap thickness and vascularization of the flap.

Recently, non‐ionizing and chairside ultrasound have been used to evaluate post‐surgical flap perfusion changes in dentistry.^23^, ^24^ Therefore, the aim of this case report is to investigate tissue perfusion changes of a GBR superficial split thickness flap procedure with ultrasound.

## MATERIALS AND METHODS

### Study design

**FIGURE 1:**
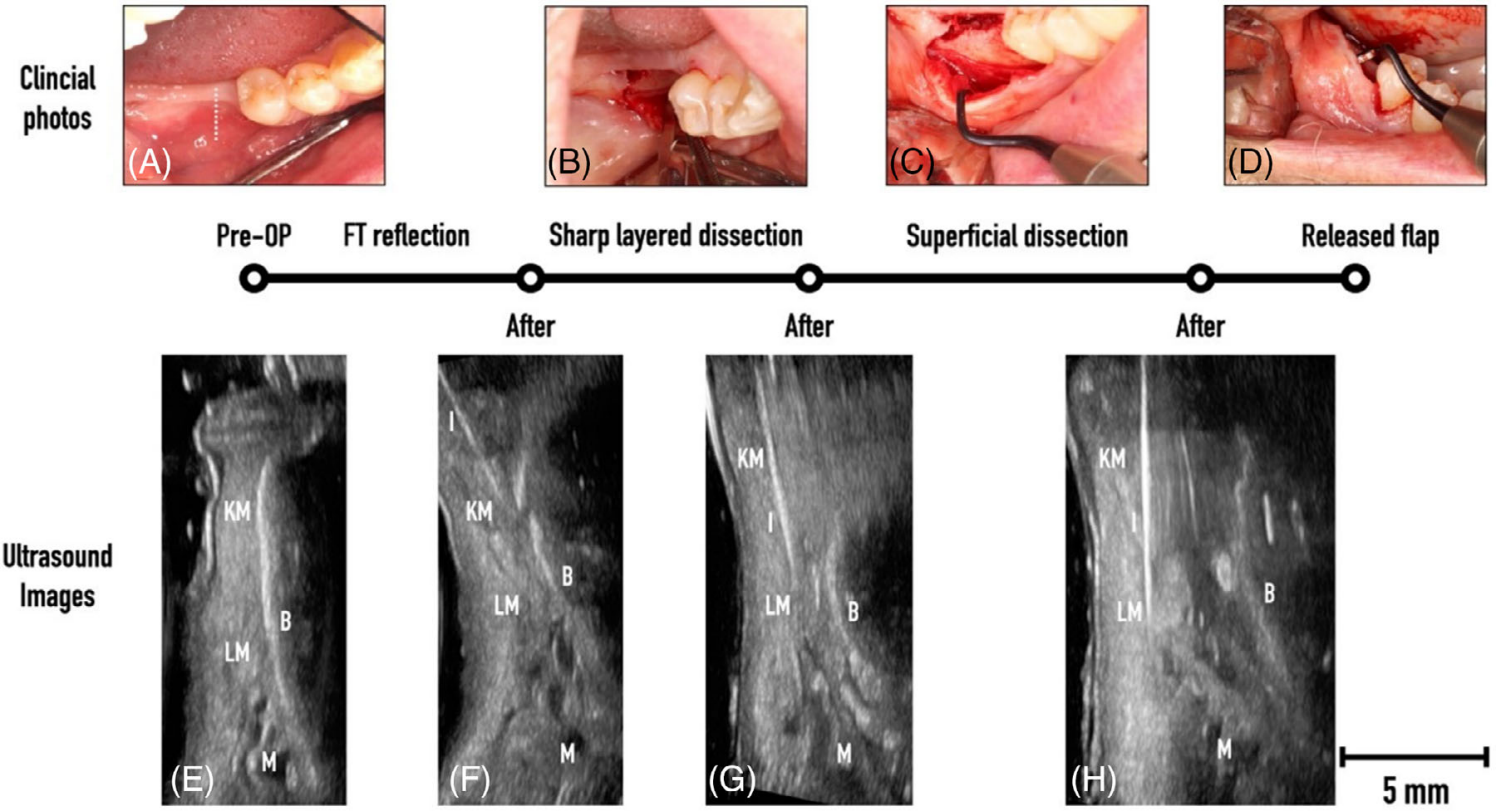
(A–D) Clinical photos presentation during the surgical procedure. (A) Presurgical occlusal view highlighting the horizontal alveolar ridge deficiency. The white dotted line indicates the orientation of the ultrasound scan. (B) Superficial linear incision of 0.5 mm deep. (C) Process of detaching the overlying mucosa from the underlying muscle fibers using a blunt instrument. (D) The released flap after the superficial split technique. (E–H) Cross‐sectional anatomical US scans of the vestibular bone ridge view, where the 3 anatomical soft regions are clearly defined: KM, keratinized mucosa; LM, lining mucosa; and M, muscle. (E) Pre‐operative scan. (F) The tip of the instrument [I] is visible as a hyperechoic straight line stopping on the alveolar bone at the level of MGJ, indicating full‐thickness reflection. (G) After the sharp layered linear incision of 0.5 mm deep, the instrument sits around 0.5 mm from the alveolar bone surface, indicating partial thickness reflection. (H) After blunt dissection, the instrument is between the submucosa and muscle layer, indicating superficial split thickness release.

**FIGURE 2:**
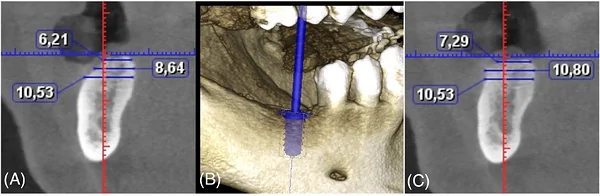
Cone beam computed tomography (CBCT). (A) Baseline measurements made at 1, 3, and 5 mm beneath the bone crest. (B) 3D representation revealed bony dehiscence if no bone augmentation was performed. (C) 5MO follow‐up measurements made at 1, 3, and 5 mm beneath the bone crest showing an increase in ridge width.

This case report was part of a randomized controlled trial approved by the University of Michigan Medical School Institutional Review Board (HUM00161016) in accordance with the Helsinki Declaration of 1975, as revised in 2013. This trial compared the clinical and radiographical healing outcomes between an active human dehydrated amnion chorion membrane* in an open wound approach and a passive collagen membrane† in a closed approach. This case report was written in accordance with CARE reporting guidelines. Written informed consent was obtained from all participants, including the patient in this case report. This case was selected due to comprehensive documentation, including step‐by‐step photographs and ultrasound images of the superficial split flap releasing procedure (SAFE technique).^25^ The patient was a 78‐year‐old male without a significant medical history, presenting at the University of Michigan School of Dentistry in 2022. The study site was the edentulous ridge #30 location, with Seibert Class 1 ridge deficiency (Figure 1A,E), planned for a staged GBR procedure, followed by single implant restoration. Cone beam computed tomography (CBCT) scan revealed bone widths of 6.21, 8.64, and 10.53 mm at 1, 3, and 5 mm beneath the bone crest, respectively. Even though the bone width seems adequate, virtual implant placement on CBCT revealed facial bony dehiscence if no bone augmentation was performed (Figure 2). 5MO CBCT was taken to evaluate the amount of ridge width gain.

### Surgical technique description

**FIGURE 3:**
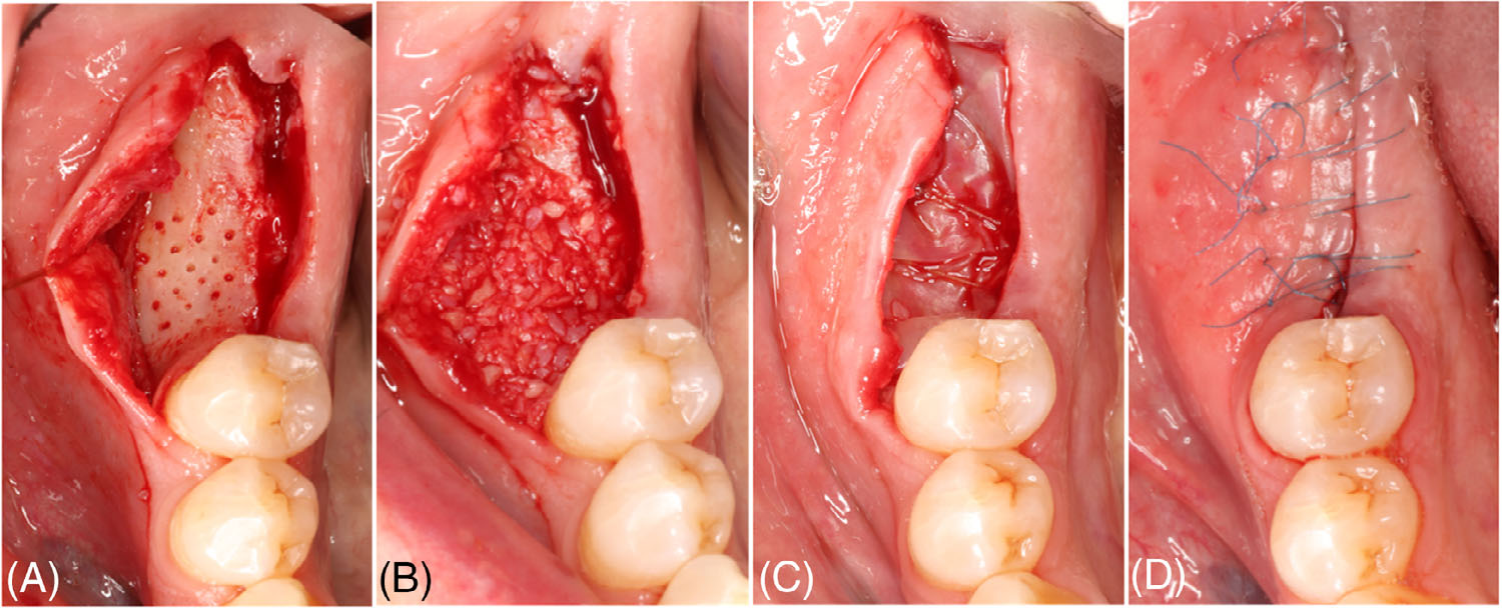
Clinical photos in chronological order from A to D: (A) The ridge surface was decorticalized. (B) The bone allograft was appropriately positioned. (C) Fixation of the human dehydrated human amnion chorion membrane was achieved using chromic gut suture. (D) The wound was closed with 7‐0 polypropylene suture.

The surgical design, rationale, and potential benefits were detailed in the original manuscript.^25^ In brief, after block anesthesia of the inferior alveolar nerve and long buccal nerve, a facially oriented incision was made following the edentulous area and in the adjacent tooth intrasulcularly.^26^ No infiltration anesthesia was given until after flap release to minimize vasoconstriction that could affect ultrasound image interpretation. A full‐thickness reflection was performed in the KM, extending 1–2 mm beyond the mucogingival junction (MGJ) (Figure 1F). A 0.5 mm linear incision was made to separate the periosteum and part of the submucosa from the flap (Figure 1B), trying to keep most of the soft tissue within the flap, thus maintaining thickness and preserving blood supply (Figure 1G). This incision extended mesially to the adjacent tooth and distally to the vertical incision close to the retromolar pad. A tunneling instrument was used to bluntly detach the overlying mucosa from the muscle fibers (buccinator muscle) using waving movements toward the facial and coronal aspects (Figure 1C,D,H). At the adjacent tooth, a full‐thickness tunneling reflection was performed. The remaining attached soft tissue was then pushed apically using a periosteal elevator to expose the native bone adequately. Particulate bone allograft‡ was placed (Figure 3B) and covered by a human dehydrated amnion chorion membrane*. The membrane was fixed to the facial periosteum utilizing resorbable 5‐0 chromic gut sutures§ (Figure 3C). The wound was closed with 7‐0 polypropylene sutures** (Figure 3D).

Postoperative medications include amoxicillin 500 mg TID for 7 days, ibuprofen 600 mg Q4‐6 h PRN for pain, and dexamethasone 2 mg nine tabs tapered (four tabs STAT, two tabs for 2 days, and one tab the last day).

### Ultrasonography and data analyses

**FIGURE 4:**
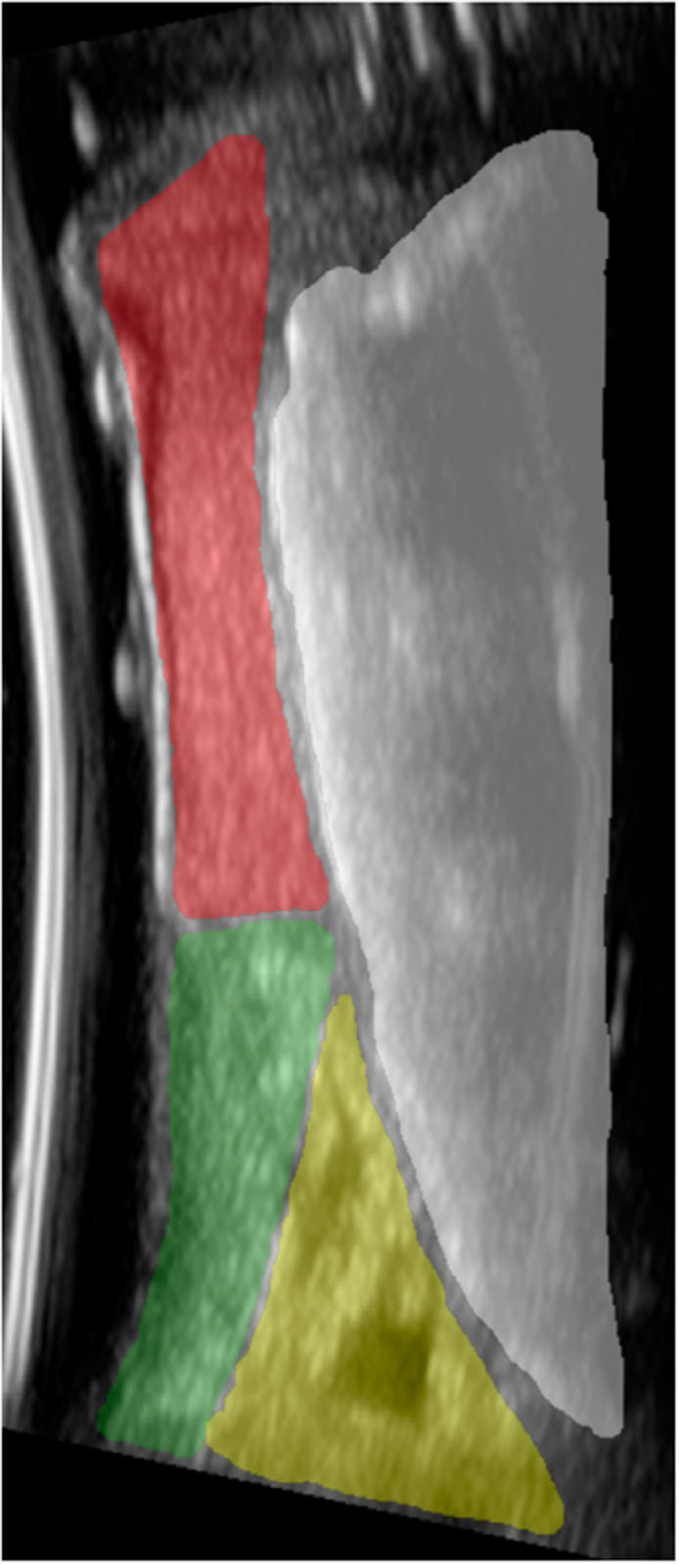
Cross‐sectional anatomical scan of the alveolar ridge. The sound wave reflects strongly at the bone surface, beyond which there are little or no reflected signals. Therefore, the bone is depicted in white color. In the soft tissue, this ultrasound b‐mode image highlights three different anatomical regions of interest: (1) The keratinized mucosa (KM), characterized by a bright (hyperechoic) surface (salmon color), (2) the lining mucosa, which is apical to the KM with a less bright surface (green color), and (3) the muscle fibers with hypoechoic, striated features (yellow color).

Ultrasound scans were conducted at baseline (BL), days (D) 3, 10, and 21, and 5 months (MO) postoperatively. Color flow images contain information about blood flow velocity and the relative amount of red blood cells within the ultrasound beam in the received signal,^27^ known as color power. Velocity and power data can be obtained as the scattered signal from moving red blood cells, as long as there is a finite projection of the actual flow direction onto the ultrasound beam.^28^ Color pixel density is given by the percent colored pixels, located in a specific region of interest (ROI).^27^ Each pixel was matched with the color bar displayed in each image; color pixels and their color hue were identified by custom software††, which translates color hue to velocities according to the present velocity scale. Red colors signify that blood flow is flowing towards the transducer; in contrast, blue color indicates flow in the opposite direction.^24^, ^27^, ^28^ Color power is shown as a single hue of red and is frequently used to detect flow in small vessels or low‐velocity flow situations.^23^, ^29^, ^30^, ^31^ B‐mode, color velocity (CV), and color power (CP) assessments were performed at the buccal side. Still images and six‐second cine loops/videos capturing at least five cardiac cycles were acquired for each visit. To quantify tissue perfusion by surrogate, cine loops (videos) were recorded for CV and CP. Ultrasonography measurements were taken on B‐mode images using a commercially available software package‡‡, as previously described. Three ROIs were defined in the facial soft tissue (Figure 4): the keratinized mucosa (KM), the lining mucosa (LM), and the muscle layer (M). KM can be identified by a thick hyperechoic line on the external surface^32^ with a hypoechoic line located underneath, which anatomically corresponds to the rete‐pegs.^33^ In contrast, LM was characterized by the lack of a hyperechoic line, and M was identified as a hypoechoic structure with internal striations that was located 8.2 mm apical to the crest of the bone.^34^ Velocity (speed) and power weighted color pixel densities were computed from CV and CP using custom scripts^¶^ by Oliver Kripfgans. Mean pixel densities (*CPD*), either velocity or power weighted, are calculated as:

$$CPD_{vel}=\sum_{i\in R}\left|v_{i}\right|dS_{i}/\sum_{i\in R}dS_{i}CPD_{pwr}=\sum_{i\in R}p_{i}dS_{i}/\sum_{i\in R}dS_{i}.$$

Here, *v_i_* and *p*_i_ are the individual ROI pixel values for CV and CP, respectively. Each pixel occupies an area of *dS_i_*.

### Clinical wound healing evaluation using the MAPS index

The MAPS healing index assessed clinical wound healing following GBR.^35^ This index aims to predict the outcome of hard tissue healing by incorporating four categories of essential factors related to flap health: bio **m** echanics, **a** esthetics/anatomy, **p** athophysiology, and **s** ubject‐specific variables. These were assessed at D3, D10, D21, and 5MO post‐surgery.

### Statistical analysis

Descriptive statistics (mean values and standard deviations) describing changes in blood flow over time were employed. Mean values averaged the pixel density from CV and CP over 6 s (approximately 100 image frames). T‐tests and linear regressions compared blood flow in three regions (KM, LM, and M) and across 5 time points (BL, D3, D10, D21, and 5MO), respectively. The significance value (*p*) was set at 0.05.

## RESULTS

Five months following the procedure, a CBCT scan was conducted, showing bone widths of 7.29, 10.80, and 10.53 mm at 1, 3, and 5 mm beneath the bone crest, respectively. By superimposing the two sets of CBCT scans, bone widths increased 1.08 and 2.16 mm at 1 and 3 mm depth, respectively (Figure 2C).

### Clinical wound healing evaluation using the MAPS index

**FIGURE 5:**
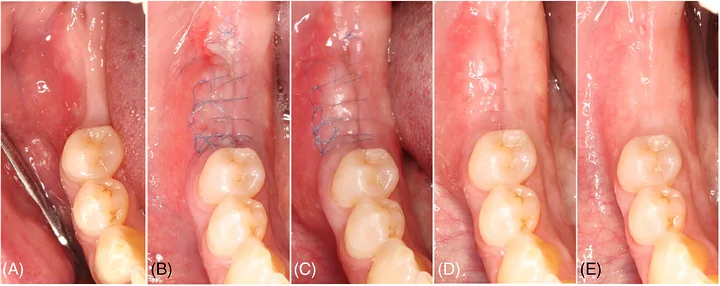
Clinical photos at (A) baseline, and (B–E) 3, 10, 21 days, and 5 months follow‐ups, demonstrating uneventful wound healing and increased ridge volume.

**FIGURE 6:**
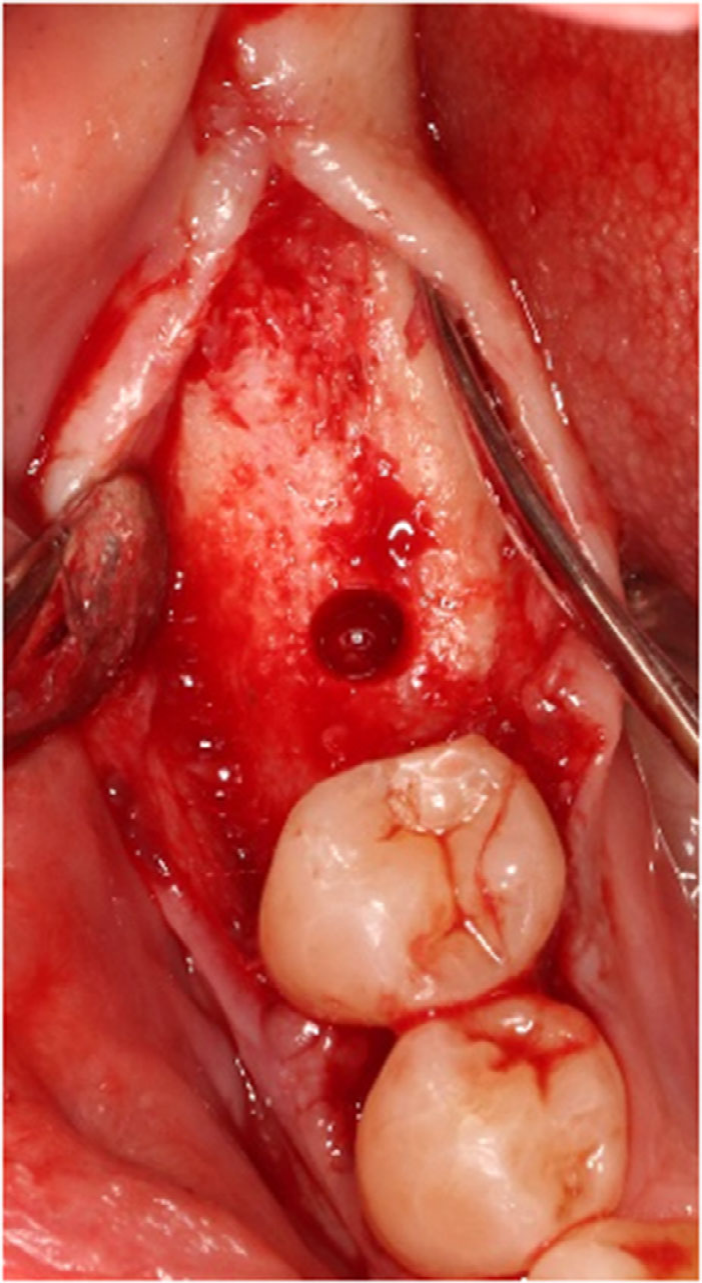
Clinical photo at implant surgery visit at 6 months after regenerative procedure, showing adequate ridge width for a standard implant.

Table 1 summarizes the MAPS healing scores. The mean MAPS scores showed an improving trend at D3 (2.9/total 4 points), D10 (3.0), D21 (3.5), and 5MO (3.8), respectively. These findings indicate a generally optimal soft tissue wound closure, resulting in clinical resolution of inflammation, enhancement in the soft tissue tone, and eventual volume improvement of the edentulous ridge (Figure 5). Osteotomy was performed for implant placement, showing sufficient bone width (Figure 6).

**TABLE 1:** Clinical wound healing evaluation using MAPS index (4 scores for each of the 4 categories, a higher score in general means better healing).

Domains	Criteria	3 days	10 days	21 days	5 months
Mechanical	Wound edge approximation	2: open wound margins but not full thickness	3: edge to edge with visible incision line		
Anatomical	Quality vs adjacent tissues	2: moderate	3: minor		4: no
	Quantity vs adjacent tissues	2: moderate	3: minor		4: no
Physiological	Signs of infection	4: no exudate			
	Hemostasis	3: fibrin at incision	4: no fibrin		
	Tissue perfusion	4: no necrosis			
	Tissue color discoloration	2: moderate	3: slight		4: normal
	Epithelization	2: partially present	3: complete, though evidence of depression		
Subject	Discomfort	4: no			
	Habit change / function impairment	4: no			
Average points		2.9	3	3.5	3.8

### CV/Power analysis

**FIGURE 7:**
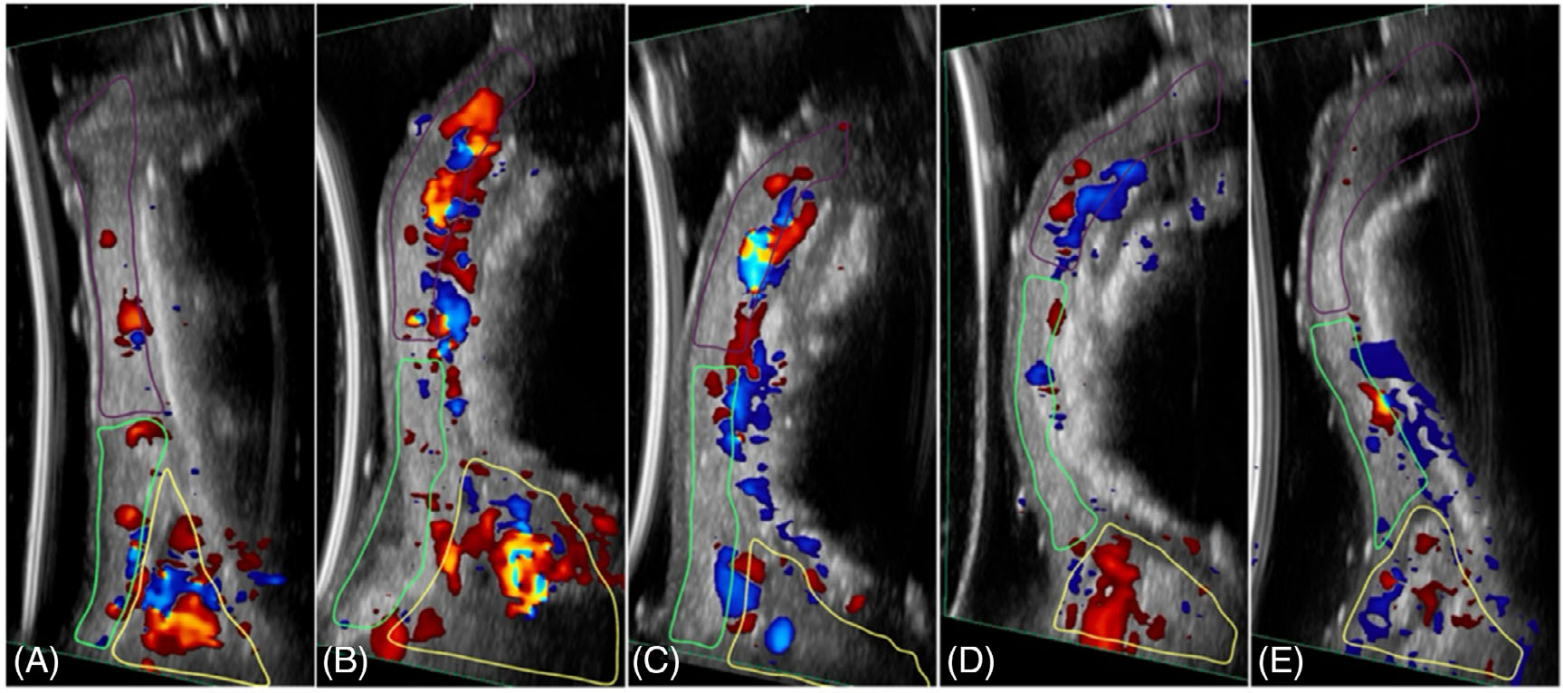
Ultrasound color flow velocity from baseline to 5‐month post‐op visits. Color pixels indicate per‐pixel mean blood flow velocity with respect to the transducer. Red and blue correspond to positive and negative velocities, i.e., to and from the transducer, respectively. (A) The baseline image shows sporadic blood flow in the keratinized mucosa (KM) (purple region of interest [ROI]) as well as lining mucosa (LM) (green ROI), with significant flow in the muscle layer (yellow ROI) compared to the KM, which might be the branches of mental vessels. (B) A similar pattern was observed on Day 3, KM displayed a significant increase in blood flow velocities, while the other two ROIs showed moderate increases. (C) On Day 10, significant blood flow was still present in KM. (D) and (E) Color flow velocity diminished progressively over time, to eventually return to baseline.

**FIGURE 8:**
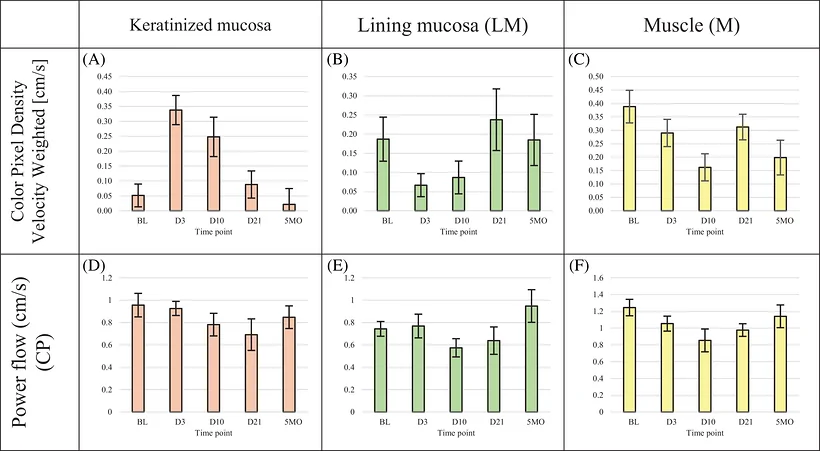
Bar graph with color flow velocity and power values. The abscissa represents visitation time points, while the ordinate reflects color pixel density values expressed. (A) CV: post‐surgical values for KM exhibited a significant increase compared to baseline, followed by a gradual decline in subsequent visits. (B) CV: LM values significantly decreased at day (D) 3 and D10, before rising again at D21 and 5MO to returning to baseline. (C) CV: Muscle values exhibited a non‐significant decline from BL to 5MO. (D‐F) CP values did not significant patterns over the healing timeline.

**FIGURE 9:**
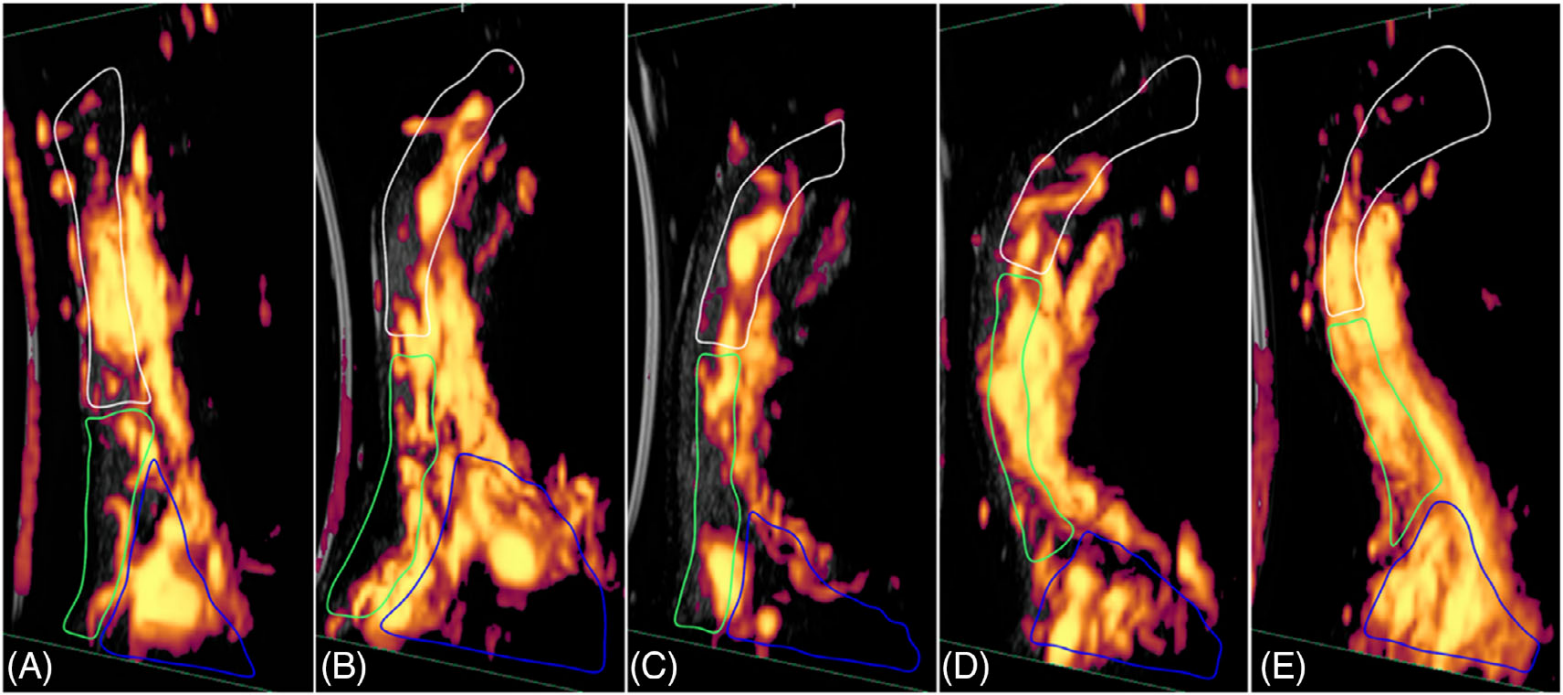
Color flow power images shown across five time points of the healing period. Red to yellow color hues indicate the amount of moving blood within each pixel. (A) At baseline, power occupies a significant area in the three zones. (B) to (E), Day 3, Day 10, Day 21, as well as 5 months, there is no apparent trend in color power values.

Quantitative results of velocity and power images are summarized in Tables 2, 3, 4. Color flow velocity images (Figure 7) showed significantly increased values in KM at D3, gradually decreasing at D10, returning to baseline at D21. LM values decreased significantly at D3 and D10, then rose slightly at D21 and 5MO. M showed inconsistent patterns with a general decreasing trend (Figure 8). Color power images (Figure 9) revealed no significant trend across the healing time (Figure 8). Further cases may be needed to clarify whether blood flow patterns bear temporal correlations to wound healing.

**TABLE 2:** Color flow velocity values (cm/s) of three tissue regions of interest (ROI, KM: keratinized mucosa, LM: lining mucosa, and M: muscle); and across the 5 time points (BL: baseline, D3: day 3, D10: day 10, D21: day 21, 5MO: 5 months). For each combination, mean ± standard deviation across at least 5 cardiac cycles is given.

ROI	BL	D3	D10	D21	5MO
KM	0.05 ± 0.04	0.34 ± 0.05	0.25 ± 0.07	0.09 ± 0.05	0.02 ± 0.05
LM	0.19 ± 0.06	0.07 ± 0.03	0.09 ± 0.04	0.24 ± 0.08	0.18 ± 0.07
Muscle	0.39 ± 0.06	0.29 ± 0.05	0.16 ± 0.05	0.31 ± 0.05	0.20 ± 0.06

**TABLE 3:** Color flow power values (arbitrary units) of three tissue regions of interest (ROI, KM: keratinized mucosa, LM: lining mucosa, and M: muscle); and across the 5 time points (BL: baseline, D3: day 3, D10: day 10, D21: day 21, 5MO: 5 months). For each combination, mean ± standard deviation across at least 5 cardiac cycles is given.

ROI	BL	D3	D10	D21	5MO
KM	0.96 ± 0.11	0.93 ± 0.06	0.78 ± 0.10	0.69 ± 0.14	0.85 ± 0.10
LM	0.74 ± 0.07	0.77 ± 0.11	0.57 ± 0.08	0.64 ± 0.12	0.95 ± 0.15
Muscle	1.25 ± 0.10	1.05 ± 0.09	0.85 ± 0.14	0.98 ± 0.08	1.14 ± 0.14

**TABLE 4:** Color velocity and color power changes over 5 months. Percentage differences between the baseline (BL) and follow‐up visits (D3: day 3, D10: day 10, D21: day 21, 5MO: 5 months).

CDV		PDI	
Tissue type/Time points	Values relative to BL	Tissue type/Time points	Values relative to BL
KM		KM	
D3‐BL	555.9%	D3‐BL	−3.1%
D10‐BL	381.2%	D10‐BL	−18.2%
D21‐BL	71.1%	D21‐BL	−27.6%
5MO‐BL	−58.1%	5MO‐BL	−11.3%
*p*‐value	***0.01***	*p*‐value	0.6
LM		LM	
D3‐BL	−64.2%	D3‐BL	3.4%
D10‐BL	−53.5%	D10‐BL	−22.8%
D21‐BL	27.2%	D21‐BL	−14.2%
5MO‐BL	−1.1%	5MO‐BL	27.5%
*p*‐value	0.2	*p*‐value	0.5
M		M	
D3‐BL	−25.3%	D3‐BL	−15.4%
D10‐BL	−58.3%	D10‐BL	−31.5%
D21‐BL	−19.6%	D21‐BL	−21.5%
5MO‐BL	−48.9%	5MO‐BL	−8.3%
*p*‐value	0.8	*p*‐value	0.6

In more detail, KM revealed a significant difference (*p* < 0.01) (Table 4) for CDV changes over time compared to BL. A difference of 556% was found between BL and D3, decreasing to 381% at D10, 71.1% at D21, and reaching a 5MO value of ‐58.1% relative to BL. No significant differences were found by the linear regression for LM and M over time, i.e., *p* = 0.2 and *p* = 0.8, respectively. LM showed reduced perfusion by ‐64.2% and ‐53.5% at D3 and D10, respectively, rebounding at D21 by 27.2%. M showed a non‐monotonic decrease post‐surgery. It specifically decreased at D3 (‐25.3%) and D10 (‐58.3%), then increased at D21 (‐19.6%), and then dropped again at 5MO to ‐48.9% compared to BL (Table 4).

**FIGURE 10:**
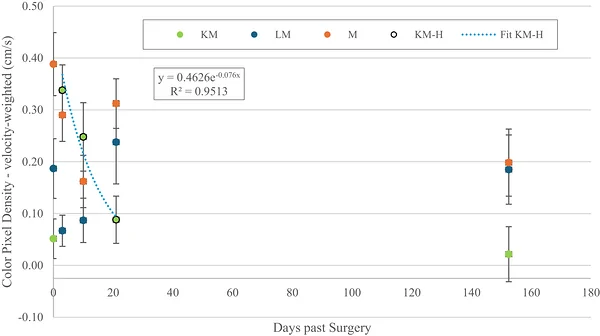
Perfusion is assessed by using color pixel density as a surrogate in three tissue types (KM: keratinized mucosa, LM: lining mucosa, M: muscle). KM shows an exponential decrease over time, as evidenced by the fit function results. LM and M did not show a systematic behavior. Note that only days 3, 10, and 21 were assessed for the fit as indicated by the open circles.

The temporal change of KM was approximated with an exponential function (y = α×e^−βx^) using days D3, D5, and D21 (KM‐healing in Figure 10), yielding fit coefficients α = 0.4626 and β = ‐0.076 (*R* ^2^ = 0.9513). The 5MO timepoint was left out since it would bias the fit by introducing a seemingly delayed recovery. According to this approximation, it would take 8.1 days for perfusion (i.e., CDV values) to reduce to 50% with respect to day 0. Furthermore, it takes 33.4 days to return to normal (0.037), which we computed as the average of BL and 5MO (see Table 2).

Linear regression analysis revealed no significant differences in PDI changes between BL and follow‐up time points (Table 4). KM fluctuated between ‐3.1% and ‐27.6% relative to BL and exhibited no statistical temporal change (*p* > 0.57). LM and M also fluctuated, namely, ‐22.8%–27.5% and ‐31.5%–8.3%, respectively. Neither LM nor M exhibited statistically significant temporal change, i.e., *p* > 0.53 and *p* > 0.59, respectively (Table 4 and Figure 8). LM nor M fluctuated between ‐64.2% – 27.2% and ‐58.3%–19.6%, respectively.

## DISCUSSION

This case report provides preliminary evidence that the “SAFE” technique is feasible to perform without jeopardizing flap vitality. Moreover, this study established a proof‐of‐concept method to estimate tissue perfusion in KM, LM, and M, revealing potentially tissue‐dependent distinct perfusion patterns during healing.

Ultrasound results showed it took ∼33 days for perfusion to return to BL. In alignment with our findings, Milstein et al., using orthogonal polarization spectral (OPS) imaging in humans, found that blood flow perfusion returned to baseline approximately 4 and 5 weeks following implant placement in conjunction with GBR.^36^ Another study, using laser speckle contrast imaging to analyze gingival vascularity after mucogingival surgery, observed that blood flow returned to baseline at 14 days.^37^ Fazekas et al., also employing laser speckle, assessed gingival blood flow after a dental extraction and implant placement.^38^ Their findings revealed a peak in KM perfusion between 7 and 14 days, with a return to baseline after 1 to 2 months. In another human study, laser Doppler flowmetry was used to evaluate gingival blood flow changes at the lining mucosa, and buccal and palatal papillae, following periodontal access flap surgery.^39^ Lining mucosa blood flow peaked on day 1, remained elevated until day 7, and returned to baseline by day 15. Blood flow in buccal and palatal papillae peaked on day 7 and normalized by day 15.

Tissue perfusion may vary depending on the performed procedures. One study showed simplified papilla preservation surgery may have a faster recovery than modified Wilderman flap surgeries.^40^ A significantly higher perfusion was observed in the coronally advanced flap (CAF) at 1 week than in the tunneling technique.^24^ In addition, ultrasonography was applied for diagnosing peri‐implant diseases.^29^ The findings revealed a significant increase in soft tissue perfusion in implants affected by peri‐implantitis, with values increasing from 200% to 300% in comparison to healthy implants. Tissue‐, procedure‐, as well as disease‐specific perfusion patterns, could lead to enhanced understanding of oral wound healing, identification of pathological development, and advances in diagnostic science.

### Limitations

The primary limitation of the study rests on its case‐report nature. The membrane used in this study might have biological effects, which may affect tissue perfusion. Future trials with larger sample sizes are necessary to validate the superficial split technique's efficacy and assess its impact on microvascular dynamics, compared to conventional methods. Methodology development for ultrasound to differentiate whether the increased blood flow is due to tissue proliferation or a pathological response is urgently needed. Measurement variability is possible due to inconsistent sampling during assessments. A reference guide could be helpful for reproducible data collection. Finally, ultrasound technology requires specific training for effective implementation.

## CONCLUSION

This case report suggests that the superficial split flap design for GBR maintains tissue perfusion as assessed with high‐frequency intraoral ultrasound. Ultrasound is feasible to quantify tissue perfusion in three anatomical structures: keratinized mucosa, lining mucosa, and muscle layers. These pilot ultrasound data ease the concern that this superficial split flap design compromises tissue perfusion. Once confirmed by larger controlled studies, ultrasound may offer a valuable tool for monitoring oral wound healing and guiding treatment decisions.

## AUTHOR CONTRIBUTIONS

Oscar Durán‐Garnica contributed to data analysis, data interpretation, manuscript preparation, and final approval of the manuscript. Karen Villarreal‐Arizpe contributed to data interpretation, manuscript preparation, and final approval of the manuscript. Amanda Rodríguez contributed to data collection, manuscript preparation, and final approval of the manuscript. Oliver Kripfgans contributed to the conception of the work, data analysis, funding acquisition, manuscript preparation, and final approval of the manuscript. Hsun‐Liang Chan contributed to data collection, analysis, funding acquisition, interpretation, manuscript preparation, and final approval of the manuscript.

## Data Availability

The data that support the findings of this study are available upon request from the corresponding author. The data are not publicly available due to privacy or ethical restrictions.

## Footnotes

* BioXclude, Maxxeus, Kettering, OH, USA

† ORAMEM, Salvin, Charlotte, NC, USA

‡ Maxxeus Bone Allograft Particulates, Maxxeus, Kettering, OH, USA

§ Ethicon, Henry Schein, Melville, NY, USA

** Unify AD surgical, Sunnyvale, CA, USA

†† MATLAB, The Mathworks, Natick, MA, USA

‡‡ Horos, version 3.3.6, Horos Project
